# Transcriptional Profiling of Common Carp: A Microarray-Based Framework for Aquaculture Research

**DOI:** 10.3390/ijms262311240

**Published:** 2025-11-21

**Authors:** Aneta Pluta, Danielle Fletcher, Monika Karwatowicz, Ewa Paździor

**Affiliations:** 1 Department of Research Support, National Veterinary Research Institute, Al. Partyzantów 57, 24-100 Puławy, Poland; monika.karwatowicz@piwet.pulawy.pl; 2 Department of Virology and Viral Animal Diseases, National Veterinary Research Institute, Al. Partyzantów 57, 24-100 Puławy, Poland; 3 Diagnostics and Genomics Group, Agilent Technologies, 5500 Lakeside, Cheadle Royal Business Park, Cheadle, Cheshire SK8 3GR, UK; danielle_fletcher@agilent.com; 4 Department of Parasitology and Invasive Diseases, Bee Diseases and Aquatic Animal Diseases, National Veterinary Research Institute, Al. Partyzantów 57, 24-100 Puławy, Poland

**Keywords:** common carp (*C. carpio* L.), custom microarray, head kidney (pronephros), gene expression profiling, aquaculture genomics

## Abstract

The common carp *Cyprinus carpio* L. is a cornerstone aquaculture species, yet transcriptome interpretation is complicated by its paleotetraploid genome and extensive alternative splicing. A species-tailored oligonucleotide microarray was developed to deliver reproducible, gene-level expression profiling. Probe design was anchored to the SPL01 reference and implemented on an Agilent platform using a gene-level strategy that collapsed transcripts to genes, selected the longest isoform, and placed 3′-anchored 60-mer probes. The workflow incorporated embedded technical controls and a standardized two-color pipeline to ensure stable measurements across arrays. Baseline functional organization of the head kidney was defined using 614 *C. carpio* L. -*Danio rerio* orthologs and complementary enrichment tools. Coherent signatures emerged for hemoglobin-mediated oxygen transport, heme and porphyrin metabolism, antioxidant defense driven by peroxiredoxin and thioredoxin systems, including hydrogen peroxide detoxification, protease regulation through *SERPIN*, *SPINK*, and *WFDC* families, and elements of innate and humoral immunity. Targets bearing *c-Myc* motifs showed a modest positive bias consistent with ongoing hematopoiesis. These resolved baseline modules provide a reference against which infection- or exposure-induced programs such as interferon-stimulated genes, chemokines and chemotaxis, complement activation, and degranulation can be detected and quantified. The platform complements RNA-seq by offering cost-efficient, rapid, and comparable measurements suited to large cohorts and longitudinal designs. Anticipated applications include host–pathogen studies for viral and bacterial agents and the assessment of chemical contaminants in aquaculture surveillance, supporting standardized, cross-study decision-making in research and health monitoring.

## 1. Introduction

The common carp (*Cyprinus carpio* L.) is a species of major importance in aquaculture and a valued model in comparative and environmental genomics [1,2]. Transcriptome profiling in this species remains challenging due to the paleotetraploid origin of its genome and widespread alternative mRNA splicing [3,4]. Consequently, gene-level analysis is preferred for reliable interpretation, particularly in studies of immunity and stress, where effect sizes are often subtle [5,6]. Although RNA sequencing (RNA-seq) transformed transcriptomics, well-designed microarrays remain a cost-effective and reproducible option for large cohort studies, longitudinal designs, and health monitoring [7]. A fixed probe content and standardized quality-control procedures facilitate cross-experiment comparability and shorten turnaround time, which is critical for fish health surveillance. This framework is directly applicable to studies of viral and bacterial pathogens and host–pathogen interactions in aquaculture, as well as to assessments of chemical contaminants and pollutants, enabling standardized, large-cohort profiling for outbreak and exposure surveillance and response. Here, we present a species-tailored oligonucleotide microarray for *C. carpio* L. designed against the SPL01 nuclear reference genome. We demonstrate platform performance using head kidney (*pronephros*), a key hematopoietic, endocrine, and immune organ in teleosts with rigorous RNA quality control, one and two-color labeling with spike-ins, and a standardized hybridization procedure [8,9,10,11]. The resulting MIAME-compliant *C. carpio* L. microarray, together with the documented design strategy, provides a practical tool for gene-expression analyses in controlled settings and aquaculture applications.

## 2. Results

### 2.1. Dye-Balance Control Test

**Figure 1:**
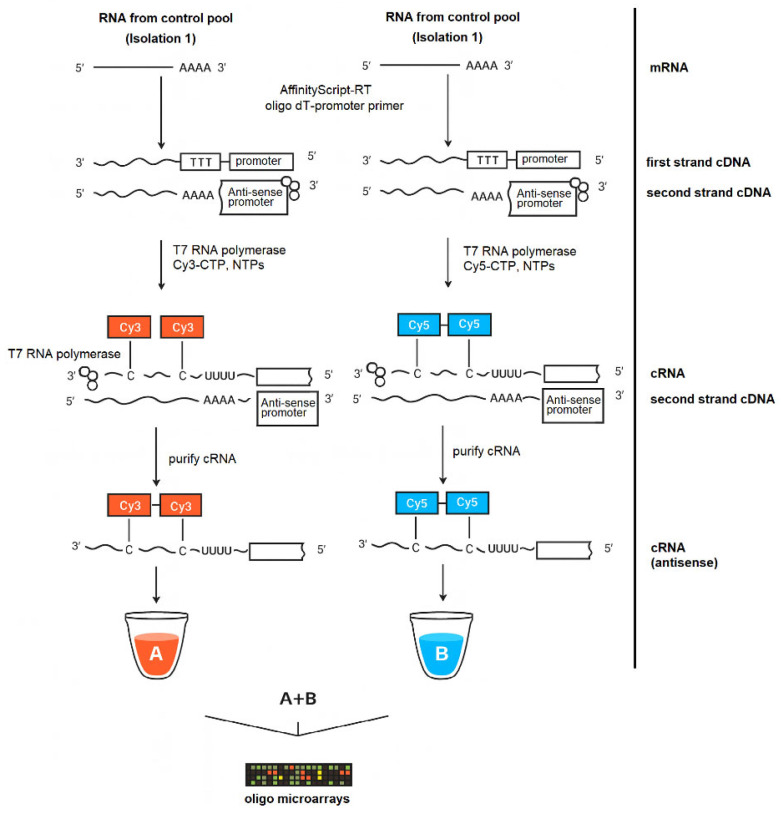
Amplified cRNA workflow for two-color oligonucleotide microarrays. Total RNA (isolation 1 control pool) was oligo(dT)-T7-primed, converted to double-stranded cDNA, and T7-transcribed to cRNA labeled with Cy3 (A) or Cy5 (B); labeled cRNA (alone or mixed A + B) was hybridized to the oligo microarray for two-color analysis (available from https://www.agilent.com/cs/library/usermanuals/public/G4140-90050_GeneExpression_TwoColor_6.9.pdf?srsltid=AfmBOoqnVSm5rjbwReMbJQ43s0-Dc4LjT_9lArxaqtHyc5o1QHFuZ_bL (accessed on 16 November 2025)).

To verify labeling consistency and exclude dye-specific bias, we performed a self-self dye-balance test using pooled control RNA co-hybridized on three subarrays with the same pool independently labeled with Cy3 and Cy5 (Figure 1). Within-array Cy5 vs. Cy3 contrasts were analyzed by a moderated *t*-test with BH correction (|FC| ≥ 2.0, *p* < 0.05). Ten probes showed significant Cy3-Cy5 differences (Figure S1), and all mapped to internal controls rather than carp transcripts, specifically members of the E1A_r60 spike-in series and DCP_22 stringency probes. As expected, E1A spike-ins exhibit defined inter-dye ratios, and DCP features report hybridization/wash stringency; neither reflects biological RNA targets. No carp specific probe displayed a significant Cy5/Cy3 imbalance, indicating balanced labeling and absence of systematic dye bias in our two-color measurements.

### 2.2. Technical Reproducibility Test: Comparison of RNA Isolation Replicates

To assess the reproducibility of RNA extraction and labeling, we compared two independent RNA isolations prepared from the same control tissue pool. Each isolation was labeled with Cy3 and hybridized on eight subarrays. Out of 62,976 probes, 12 (0.019%) showed significant differences between isolations; all were higher in Isolation 2, FC 2.07–3.88; mean log_2_FC + 1.23; Supplementary Figure S2. Annotation-based review grouped these 12 probes into clear categories. Four probes corresponded to distinct genes: *CRYGN1*, *NPY1R*, *CALY*, and *FOXP3A*, but their small, isolated shifts did not change pathway-level results.

Six probes targeted hemoglobin alpha/beta paralogs. One probe annotated as *ZP4*-like likely represents a low-abundance or technical signal in head kidney and had no impact on pathway calls. One probe annotated as a non-coding RNA cross-hybridizes to an intelectin-like transcript, which explains its isolation-2-specific increase without implying a true expression change. The array also included an intelectin-like probe that was consistently detected in both isolations and was not differentially measured. To exclude artifacts, we assessed probe-level detection, signal-to-noise ratio (SNR) distributions, and Feature Extraction (FE) flags for all 12 probes. Across all probes and 16 hybridizations, detection was complete: the present call rate was 1.0 for every probe on all arrays, and no adverse FE flags were observed (non-uniformity, population outliers, and saturation = 0 per probe). Median SNR distributions indicated strong signals above background for most probes. Extremely high SNRs were seen for the globin-family probes, consistent with abundant erythroid transcripts; several unique-gene probes showed moderate-to-high SNRs, whereas a minority of ncRNA-annotated probes exhibited lower SNRs, warranting cautious interpretation (Supplementary Table S1 and Figure S3). Together, these QC results support high confidence for the four unique targets (e.g., *CRYGN1*, *NPY1R*, *CALY*, and *FOXP3A*) and confirm that globin signals reflect family-level redundancy; ncRNA probes at lower SNR are flagged as higher cross-hybridization (x-hyb) risk and do not drive pathway conclusions.

### 2.3. Global Expression Landscape Under Control Conditions

The analysis was based on expression data from the control group, measured on 16 microarrays: 8 derived from the first RNA isolation and another 8 from a technical repetition of RNA extraction. In total, 856 transcripts were detected on the microarray, encompassing both coding and non-coding RNA species (Supplementary Table S2). Most features were annotated as messenger RNAs (mRNAs, 745 transcripts; 87.03%), with non-coding RNAs (ncRNA) accounting for 108 entries (12.62%) and three miscellaneous RNAs (miscRNA) (0.35%) were also identified. After within-array normalization by the 75th percentile method and between-array baseline transformation to the median of all samples, 602 transcripts (70.3%) displayed positive log_2_ intensities relative to the reference baseline, whereas 254 (29.7%) displayed negative values. The dominance of mRNA features suggests that the observed signal landscape primarily reflects protein-coding pathways, with non-coding classes contributing only marginally to the overall expression profile.

### 2.4. Functional Enrichment of C. carpio L.—D. rerio Orthologs (g:Profiler)

**Figure 2:**
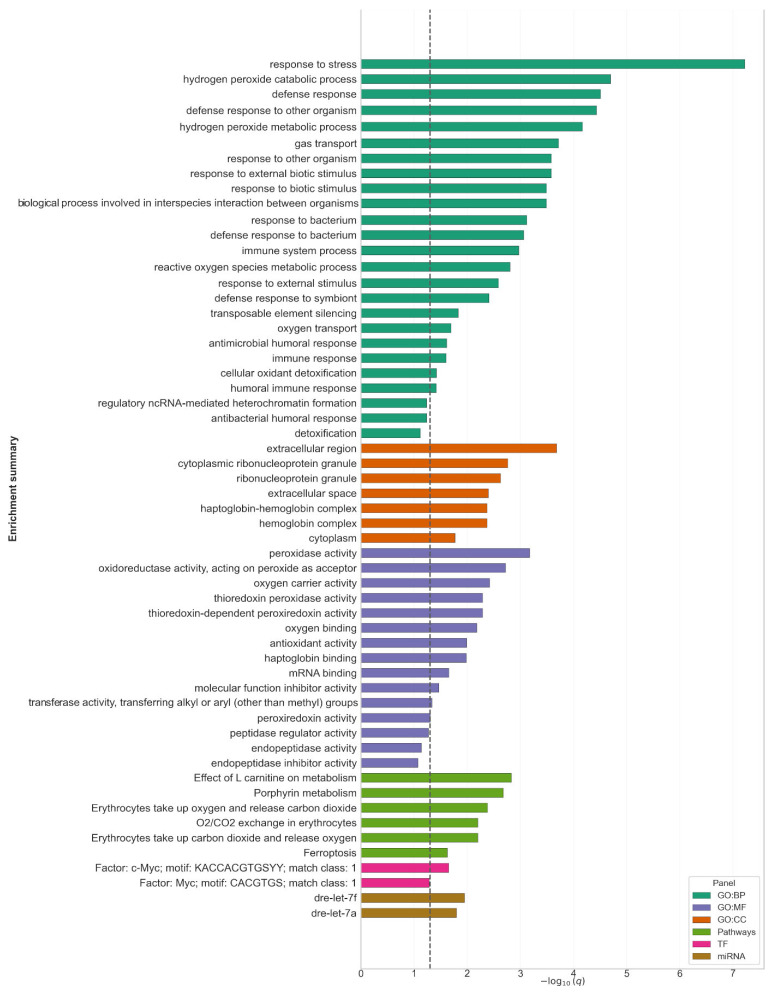
Functional enrichment of carp-zebrafish orthologs (g:Profiler). Bar plot summarizing term enrichment for 614 *C. carpio* L. genes mapped to *D. rerio* orthologs. The *x*-axis shows −log_10_(*q*), where *q* is the adjusted *p*-value; the vertical dashed line marks the significance threshold (*q* = 0.05). Bars represent the top enriched terms across annotation namespaces: GO:BP (green)-gene ontology, biological process; GO:CC (orange)-cellular component; GO:MF (purple)-molecular function; pathways (olive)-curated pathway resources (*Reactome*, *KEGG*); TF (magenta)-transcription factor motifs; miRNA (brown)-microRNA families.

For further study, 614 orthologs were retrieved (Supplementary Table S3). g:Profiler identified a coherent signature encompassing oxidative/antioxidant processes, oxygen transport, and innate immunity. Among molecular function (MF) terms, peroxidase and oxidoreductase activities were highly enriched (GO:0004601, q = 6.7 × 10^−4^; GO:0016684, q = 1.9 × 10^−3^), driven primarily by *PRDX1*/2/5 together with hemoglobin genes (*HBAA1/2*, *HBBA2*, *HBBE2*, *BA1L*, and *CYGB1*). Results are shown in Figure 2. Consistent biological process (BP) terms included response to stress (GO:0006950, q = 6.0 × 10^−8^; 66/351 hits), hydrogen peroxide catabolism/metabolism (q = 2.0 × 10^−5^–6.8 × 10^−5^; 8/351 hits), defense response (q = 3.1 × 10^−5^), and gas/oxygen transport (q = 1.9 × 10^−4^–2.0 × 10^−2^). Cellular component (CC) enrichment pointed to the extracellular region/space (GO:0005576, q = 2.1 × 10^−4^; 52/373 hits) and ribonucleoprotein granules (GO:0036464/GO:0035770, q = 1.7 × 10^−3^–2.4 × 10^−3^), while hemoglobin and haptoglobin–hemoglobin complexes were also significant (q = 4.2 × 10^−3^). At the pathway level, enrichment supported erythrocyte-related processes (Reactome: O_2_/CO_2_ exchange in erythrocytes, q = 4.1 × 10^−3^–6.3 × 10^−3^) and heme/porphyrin metabolism (KEGG: 00860, q = 2.1 × 10^−3^), with additional signals for ferroptosis (KEGG: 04216, q = 2.37 × 10^−2^) and L-carnitine metabolism (WikiPathways: WP3992, q = 1.48 × 10^−3^). Transcription-factor motif enrichment implicated Myc/c-Myc classes (q = 0.022–0.052), while miRNA enrichment highlighted dre-let-7a/7f (q = 0.011–0.016). Detailed gene lists, statistics, and complete enrichment outputs are provided in Supplementary Table S4. Overall, the enrichment profile reflects the physiology of the healthy head kidney, combining red blood cell and oxygen-transport modules with peroxiredoxin/thioredoxin antioxidant defenses and humoral/innate immune processes consistent with the organ’s hematopoietic and immune roles in teleosts.

### 2.5. Functional Enrichment Clusters (DAVID)

Using DAVID, a dominant cluster annotated as hemoglobin-mediated oxygen transport and heme/iron metabolism was identified (enrichment score = 3.52). Within this cluster, enrichment was observed for hydrogen peroxide catabolic process (GO:0004601, *p* = 1.56 × 10^−6^), peroxidase activity (MF, *p* = 3.85 × 10^−6^), and oxygen transport/binding/carrier activity (GO terms, *p* = 5.7 × 10^−6^–1.1 × 10^−5^). Pathway analyses returned Reactome terms related to erythrocyte O_2_/CO_2_ exchange (*p* = 3.2 × 10^−6^–1.2 × 10^−5^) and InterPro annotations for the globin domain (*p* ≤ 9.3 × 10^−6^). Additional enrichment was noted for hemoglobin and haptoglobin–hemoglobin complexes (*p* ≤ 4.5 × 10^−4^, FE = 13–17), consistent with a red blood cell/antioxidant axis. A second cluster involved porphyrin/heme biosynthesis (score = 2.39), including porphyrin-containing compound biosynthesis (*p* = 7.26 × 10^−4^) and protoporphyrinogen IX biosynthesis (*p* = 0.00107). Further groups comprised cellular stress responses (score = 2.33; Reactome cellular responses to stimuli/chemical stress, *p* ≤ 5.6 × 10^−3^), RNA-binding proteins (score = 2.27; GO MF RNA binding, *p* = 0.0013), and a thioredoxin-peroxiredoxin redox module (score = 1.86), reinforcing the peroxide-detoxification signal. An iron-handling cluster (score = 1.82) featured KEGG ferroptosis (*p* = 2.39 × 10^−4^) and iron uptake/transport (*p* = 8.64 × 10^−4^). Immune-related clusters covered neutrophil degranulation/innate immunity (score = 1.81) and chemokine signaling/chemotaxis (score = 1.69; IL-8-like domains; chemokine activity, *p* = 0.002–0.003). Smaller, yet coherent, clusters involved fibronectin type-II/kringle ECM features (score = 1.69), serine protease inhibitors (1.57), aquaporins/MIP channels (1.42), and nucleoplasmin/chromatin remodeling (1.30). Full term lists, statistical parameters, and gene memberships are provided in Supplementary Table S5.

### 2.6. Cross-Term Summary Mapped to Microarray Intensities

#### 2.6.1. Functional Category Trends from g:Profiler

**Figure 3:**
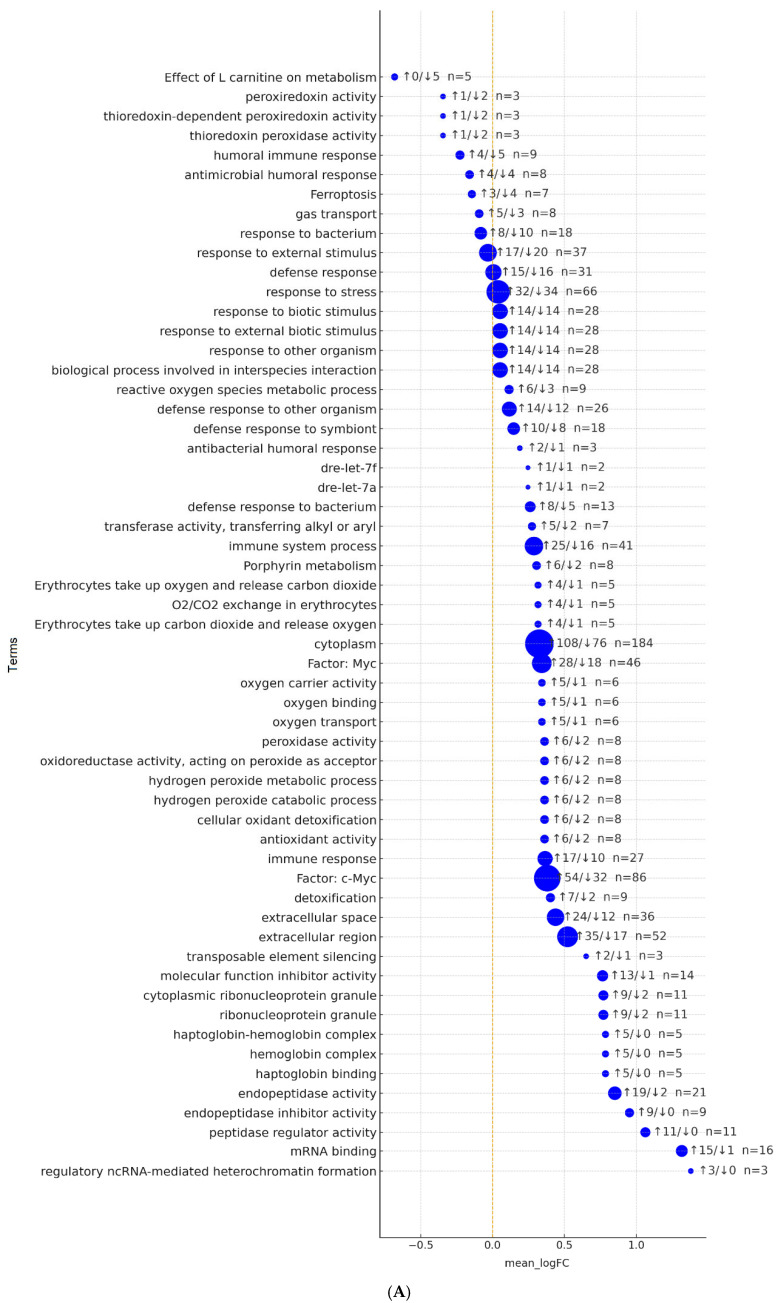

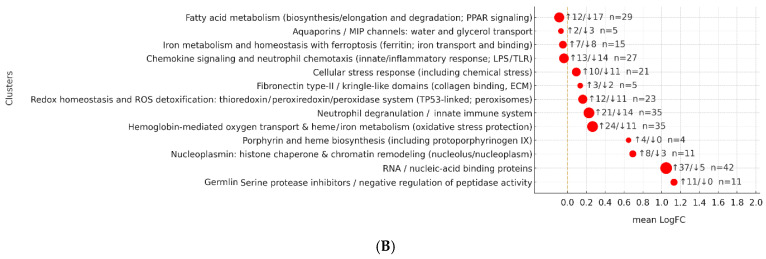
(**A**,**B**). Summary of expression changes across functional categories. (**A**,**B**) Dot plots summarize mean log_2_ fold-change (mean LogFC) values for genes grouped by functional terms or clusters. The *x*-axis represents the mean LogFC (dashed line = no directional change). Dot size is proportional to the number of genes within a given category (n), and labels indicate the counts of up and downregulated genes in the format “↑u/↓d—n”. (**A**) g:Profiler enrichment analysis: each blue dot represents a functional term (GO/pathway/motif). Terms are ordered by mean LogFC. (**B**) DAVID functional clusters: each dot corresponds to one functional cluster, sorted by mean LogFC (lowest at top). Red dots represent clusters; labels show numbers of up and downregulated genes and total gene counts.

RNA-handling terms showed strong positive shifts: mRNA binding was upregulated in 15/16 genes (mean logFC = +1.32), and cytoplasmic/ribonucleoprotein granules were up in 9/11 (Figure 3A). Protease-control modules increased; peptidase regulator and endopeptidase inhibitor activity were upregulated in all detected members *SERPIN/SPINK/WFDC*, while endopeptidase activity rose in 19 genes (2 down). Red blood cell/hemoglobin functions increased on average (mean logFC = +0.32–+0.79) across haptoglobin binding, hemoglobin complex, oxygen binding/transport, and erythrocyte gas-exchange pathways (Reactome O_2_/CO_2_ exchange). Oxidative-stress categories driven by globins and peroxiredoxins also increased (peroxidase/antioxidant activity; H_2_O_2_ catabolism/detoxification; typically 6 up/2 down per term). Immune signals were mixed and modest: immune system process 25 up/16 down (mean = +0.29) and defense response to bacterium 8 up/5 down, alongside small negative shifts in aggregate categories such as defense response, response to stress, and response to external stimulus (median = −0.60). TF-motif summaries favored *c-Myc/Myc* targets (majorities upregulated; mean logFC = +0.34–+0.38). Lipid/iron-redox modules were lower overall: ferroptosis 3 up/4 down (median = −0.69) and the Effect of L-carnitine on metabolism set 0 up/5 down (median = −0.69). Peroxiredoxin-specific terms (thioredoxin-dependent peroxiredoxin; peroxiredoxin activity) also showed slightly negative averages (1 up/2 down; median = −0.82). Overall, the control transcriptome shows increased RNA handling, extracellular/protease regulation, hemoglobin-mediated oxygen transport, and generic antioxidant programs, with mild, mixed shifts in immune and stress-response categories (Supplementary Table S6).

#### 2.6.2. Functional Cluster Trends from DAVID

The strongest positive signals involved hemoglobin metabolism and gas transport (Figure 3B). Cluster 1 (hemoglobin-mediated O_2_ transport and heme/iron metabolism) showed 24 out of 35 genes upregulated, with a median_logFC of +0.686 (e.g., *HBAA1/2*, *HBBA2*, *HBBE2*, *BA1L*, *CYGB1*, *AQP1A.1*, *SLC4A1B*, *PRDX1/2/5*, *FTH1A*, *FTHL27/28*, *BLVRA/B*). Cluster 2 (porphyrin/heme biosynthesis) showed the same pattern, with all four genes upregulated (median logFC = +0.633; *CPOX*, *HMBSA/B*, *PPOX*). RNA-handling and nucleic-acid binding functions were also prominently elevated: cluster 4 had 37 of 42 genes upregulated (median +1.183). Protease control was strongly increased: cluster 10 (*SERPIN/SPINK/WFDC* family; negative regulation of peptidase activity) showed all 11 genes upregulated (median +0.924). Within innate immunity, cluster 7 (neutrophil degranulation/innate immune system) skewed positive (21/35 up; median +0.615), while extracellular matrix/adhesion features were modestly increased (cluster 9, 3/5 up; median +0.714). Oxidative-stress modules were heterogeneous: cluster 5 (thioredoxin-peroxiredoxin/peroxidase) was near-balanced (12/23 upregulated; median logFC = +0.607), whereas the broader cellular stress response (cluster 3) trended slightly negative (10/21 upregulated; median logFC = −0.605). Iron handling with a ferroptosis component was mildly reduced (cluster 6, 7/15 up; median −0.605). The most consistent decreases involved chemokine signaling and neutrophil chemotaxis: cluster 8 showed 13/27 up vs. 14/27 down (median −0.678), including *CXCL8a*, *CXCL11.1*, *CXCR2*, *CD209*, *SAA*, and *CCL19*. Aquaporins/MIP channels (cluster 11) were slightly downregulated (2/5 up; median −0.715). Overall, healthy controls are characterized by coherent upregulation of oxygen transport/heme metabolism, RNA binding/processing, peptidase inhibition, and elements of the innate immune system, with modest downregulation in chemokine/chemotaxis, aquaporin modules, and selected lipid- and ferroptosis-linked programs (Supplementary Table S7).

### 2.7. Cross-Platform Concordance in the Healthy Control Set

**Figure 4:**
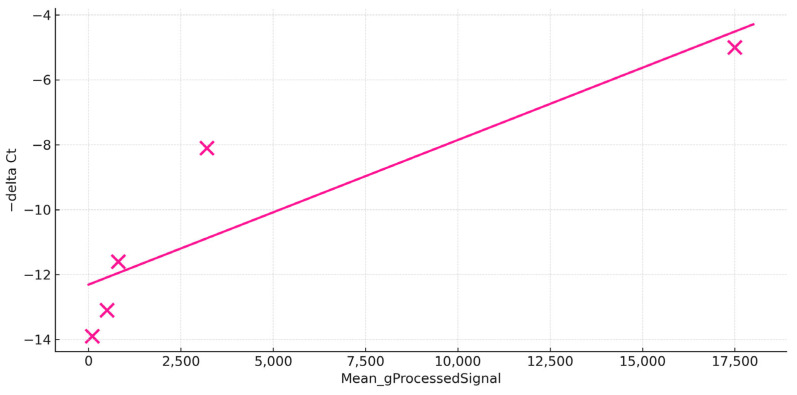
Microarray validation by RT-PCR. The scatter plot comparing quantitative RT-PCR and microarray readouts. The *y*-axis displays −ΔCt (RT-PCR), while the *x*-axis shows the microarray Mean_gProcessedSignal (microarray intensity), which is the background-corrected probe intensity. Each data point (depicted as a pink cross) corresponds to one gene (*SAA*, *CCL19*, *CD209*, *MMP9*, or *CXCL8a*). The solid line represents the least-squares linear fit, demonstrating a positive association between the two platforms when Pearson’s R = 0.896.

In the healthy control, we assessed agreement between microarray per-channel intensity (Cy3; Mean_gProcessedSignal) and RT-qPCR readouts (−ΔCt) for five targets (*SAA*, *CCL19*, *CD209*, *MMP9*, *CXCL8a*). The two platforms showed a positive association (least-squares fit: slope = 0.0004368, intercept = −12.177; Pearson R= 0.896, *n* = 5, *p*= 0.04), with ranking consistency across genes, with highest abundance by both methods for *SAA* and lowest for *CXCL8a* (Figure 4). 

These results indicate that, in the healthy group, microarray intensities capture relative transcript abundance in line with RT-qPCR measurements; note that this analysis evaluates expression level concordance within one condition, not differential expression between groups.

## 3. Discussion

This study presents a species-tailored, MIAME-compliant oligonucleotide microarray for *C. carpio* L. and demonstrates its analytical performance on healthy head kidney (pronephros) tissue. Evidence from the dye-balance control, RNA-isolation reproducibility, and RT-qPCR concordance supports stable signals with low dye bias and limited extraction-related drift. These quality controls are critical in teleosts, where a paleotetraploid genome and extensive alternative splicing amplify the risk of probe redundancy and cross-hybridization [3,4]. These features render the platform directly applicable to studies of viral and bacterial pathogens in aquaculture, enabling standardized host-response profiling during experimental challenges and field outbreaks. The design (gene-level collapsing, 3′-anchored 60-mers, cross-hybridization screening) was selected to mitigate probe redundancy and cross-hybridization, yielding gene-level readouts on the 8 × 60 K array. This study complements earlier carp genomic resources, including the cross-species array and EST set of Williams et al. [12] and the de novo transcriptome of Ji et al. [13], by providing a design explicitly tied to the modern nuclear reference SPL01 (ASM1834038v1). In contrast to earlier platforms that mixed carp and heterologous probes or targeted transcript fragments of variable provenance, the present array was built from contemporary gene models and systematically filtered for redundancy. This approach follows established guidance for teleost immunology and health surveillance, where large cohorts and standardized workflows are required [14]. Practically, the fixed probe content and embedded controls (spike-ins, DCP stringency probes) shorten turnaround time and facilitate between-study comparability, a recognized advantage of microarrays in longitudinal aquaculture monitoring [5,7]. In practical terms, the platform can support aquaculture surveillance by providing gene-expression signatures that act as early indicators of stress, inflammation, or immune activation before clinical signs are detectable. The array may also assist in pathogen monitoring by revealing characteristic transcriptional responses associated with viral or bacterial exposure, thereby complementing conventional diagnostic approaches. Furthermore, its standardized gene-level output makes it suitable for routine assessment of environmental stressors such as hypoxia, ammonia, or temperature fluctuations, facilitating decision-making in husbandry and health management.

The dye-balance test produced significant Cy3/Cy5 differences exclusively in Agilent control features (E1A spike-ins and DCP stringency probes), indicating balanced labeling and the absence of dye-specific effects on carp probes. The comparison of independent RNA isolations (8 arrays per isolation; all Cy3) identified 12 differentially measured probes. Predominance of higher signals in Isolation 2 is consistent with small, acceptable pre-analytical deviations at the isolation and/or amplification stage; in T7/oligo(dT) system, transcripts enriched toward the 3′ region can be preferentially amplified without dye effects (single channel Cy3 analysis) [15,16]. Annotation review identified four unique genes among the 12 probes: *CRYGN1*, *NPY1R*, *CALY*, and *FOXP3A*; differences for these probes did not change pathway assignments or functional conclusions because they did not form a coherent signal at the level of GO categories or pathways [17,18]. Six probes were mapped to the globin family and their close alpha/beta paralogs. In teleosts, these families are multi-gene and highly conserved, favoring family-level redundancy and single 60 nt probe may partially read the sum of very similar transcripts from different loci [19,20]. This behavior amplifies the signal of the functional module (oxygen transport/erythropoiesis) without shifting pathway-level interpretation and remains consistent with the “one-probe-per-gene” design that reduces isoform redundancy but does not eliminate effects arising from very high homology among paralogs [21,22]. Because hemoglobin genes form a highly homologous multigene family, individual 60-mer probes may capture more than one paralog. We therefore interpret these signals at the hemoglobin module/family level (oxygen transport/erythropoiesis) rather than attributing changes to a single globin gene. Two probes involved the intelectin family and explain the apparent asymmetry between isolations: probe -intelectin-like produced stable signal in both isolations, whereas the second probe -ncRNA showed high sequence similarity to a closely related intelectin-like transcript, promoting cross-hybridization and accounting for the higher signal in Isolation 2 without a true change in expression of the target gene [21,23]. The remaining two probes -ncRNA with sparse annotation and probe-*CCDC96*-like were single calls typical of near-background signal and were treated as technical or indeterminate, with no pathway consequences [24,25]. Probe-level quality control of the 12 differentially measured probes confirmed robust detection across arrays, with consistent present calls and no adverse Feature Extraction flags. Signal-to-noise distributions indicated strong signals above background for most probes, including those targeting the globin family, in line with abundant erythroid transcripts, whereas a minority of ncRNA-annotated probes showed lower specificity signals that warrant cautious interpretation. These observations support our conservative practice of summarizing highly homologous families (e.g., globins) at the module/family level rather than attributing effects to single paralogs, and of treating singleton, low-specificity calls as technical unless independently validated (e.g., paralog-discriminating RT-qPCR). For *CCDC96*-like, cross-hybridization was particularly plausible because coiled-coil motifs are long, low-complexity heptad repeats widely distributed in cytoskeletal and centrosomal proteins. Such segments allow partial matches among related transcripts and can yield stable 60-mer hybridization despite mismatches, and paralogy of these families in teleosts further accentuates the effect [26,27]. In practice, we interpret *CCDC96*-like probes as technical or family signals and recommend RT-qPCR in a unique region, and platform update an alternative probe in a more variable 3′ UTR segment or unique exon [28,29]. Elevated fold changes observed for zona pellucida sperm-binding protein 4-like (*ZP4*-like) are most plausibly explained by technical and biological factors rather than true differential expression. In fish, ZP proteins form the egg envelope and their genes are classically expressed in the oocyte and/or induced by estrogens in the liver during vitellogenesis; single ZP hits in somatic tissues typically arise from cross-hybridization to ZP-like domains in other secreted proteins, very low-level noise from homologous transcripts, occasional admixture of hepatic or plasma material, or a 3′ amplification bias [16,21,30,31]. Accordingly, single *ZP4*-like probes with |FC| > 2, without support from multiple probes or primers and without tissue concordance, should be treated cautiously as candidates for RT-qPCR verification with specific primers [28]. Sensitivity tests confirmed the robustness of functional conclusions: removing all 12 probes did not alter the ranking or substance of enrichments (hemoglobin/gas transport, porphyrin/heme metabolism, peroxiredoxin/thioredoxin antioxidant axes, and elements of innate/humoral immunity) [17,18]. Overall, the picture is expected for an allotetraploid species and a hybridization platform: rare deviations across technical repeats stem mainly from family paralogy, cross-hybridization, or near-background signals, without changing process- and pathway-level conclusions [3,21]. The “one-probe-per-gene” design, with 3′-anchored probes compatible with T7/oligo(dT), effectively reduces isoform redundancy and intra-gene cross-hybridization risk, though it cannot remove effects due to homology among multi-gene families; for such families, multi-probe variants targeting more variable 3′ regions, paralog-discriminating RT-qPCR, and systematic in silico specificity reviews are warranted [16,21,22].

The head kidney is the principal hematopoietic and an immune-endocrine interface in teleosts [8,32], and the enrichment profile is consistent with this physiology. g:Profiler and DAVID identified overlapping signals encompassing hemoglobin/oxygen transport, heme-porphyrin metabolism, antioxidant defense (peroxiredoxin/thioredoxin and H_2_O_2_ detoxification), and innate/humoral immunity [33]. The Reactome “O_2_/CO_2_ exchange in erythrocytes”, KEGG heme biosynthesis, and InterPro globin-domain signals are entirely consistent with ongoing erythropoiesis and red-cell turnover in the pronephros [9]. The strong serpin/*SPINK/WFDC* negative regulation of peptidase activity cluster fits secretory control of protease cascades in fish plasma and extracellular matrices that buffer inflammation and tissue remodeling in healthy states-patterns also seen in salmonid spleen/head kidney microarrays [14,34]. Although the present dataset consists of healthy controls, the resolved baseline modules provide a reference against which infection-induced programs, type I interferon-stimulated genes, chemokines/chemotaxis, complement activation, and degranulation can be detected and quantified during viral or bacterial challenge.

The apparent over-representation of selected processes reflects the tissue’s cellular composition and anatomy [35]. First, head kidney contains a mixture of erythroid precursors, myeloid cells, lymphoid cells, stromal elements, and endocrine cells [8]. Pathways fundamental to these compartments following the hemoglobin assembly, iron/porphyrin metabolism, redox buffering, and secretory regulation will draw from many distinct genes, inflating counts in GO and pathway terms [36]. Second, terms such as cytoplasm aggregate housekeeping and regulatory proteins from multiple lineages; conversely, specialized processes (e.g., chemokine-mediated neutrophil chemotaxis) are encoded by relatively few, lineage-restricted genes and thus appear with smaller gene counts. Finally, the gene-level probe design was chosen to reduce isoform multiplicity, so categories driven by gene families (globins, serpins, *PRDXs*) remain prominent, whereas isoform-dense categories are intentionally compressed, limiting inflation of counts [37]. The GO term response to stress was represented by 66 genes in our ortholog-mapped set, split approximately evenly between up and downregulation [38]. In a tissue that continuously executes hematopoiesis and immune surveillance, a mixed directionality is expected and indicates physiological homeostasis rather than a dominant inflammatory program. If a systemic inflammatory process were underway, stress-response and innate-immune categories (e.g., chemokines, degranulation, and interferon-stimulated genes) would skew strongly upward, as reported during pathogen challenge in salmonids [14,39]. In our healthy carp, chemokine signaling/chemotaxis trended neutral-to-negative on average, further supporting absence of ongoing inflammation.

The cellular-component term cytoplasm encompassed 184 genes (108 up, 76 down). The breadth reflects the term’s scope: it aggregates multiple functional groups operating in the cytosol. Among the 108 upregulated entries, RNA-handling and ribonucleoprotein-granule components, RNA-binding proteins, translation/elongation factors, cytosolic chaperones, and antioxidant enzymes (e.g., *PRDXs*) were over-represented, consistent with active mRNA processing and proteostasis in hematopoietic and immune cells. In contrast, the 76 downregulated cytoplasmic entries were enriched for select metabolic enzymes, cytoskeletal/trafficking elements, and scattered stress-responsive proteins whose lower averages are compatible with a resting, non-migratory state of many leukocyte subsets in healthy fish [40]. Because “cytoplasm” denotes a location rather than a process, mixed directionality is expected; the term pools pathways with distinct homeostatic set points.

The *c-Myc* motif set comprised 86 genes (54 up, 32 down). *c-Myc* is a pleiotropic transcription factor that coordinates ribosome biogenesis, nucleotide metabolism, and cell-cycle entry in hematopoietic progenitors and activated lymphocytes [41]. A moderate bias toward upregulation is physiologically consistent with ongoing erythropoiesis/leukopoiesis and tonic immune signaling [42]. The signal was not accompanied by concordant increases in inflammatory chemokines or cell-injury markers, which is inconsistent with pathological activation. The pattern is consistent with baseline proliferative and biosynthetic demands of progenitor niches and resident immune cells in the teleost head kidney [8,43].

The extracellular region (*n* = 52) and extracellular space (*n* = 36) terms were both up-biased. Head kidney produces and exports many soluble mediators, complement components, lectins, acute-phase proteins (e.g., *SAA*), protease inhibitors (*SERPINs/WFDC*), carrier proteins, and matrix regulators that sustain plasma homeostasis and shape local microenvironments [44]. Enrichment of these terms is expected in healthy fish and is consistent with reports from salmonid immune tissues under control conditions, where humoral immunity and secretory regulation remain prominent without experimental challenge [14,45]. Together with hemoglobin/porphyrin and antioxidant signatures, these extracellular terms are consistent with a head kidney that secretes plasma proteins, buffers oxidative load, and contributes to systemic proteostasis.

RNA-seq remains indispensable for the discovery of novel isoforms, allele-specific expression, and deconvolution of subgenomes in allotetraploid carp [46,47,48]. In operational aquaculture and large multi-batch studies, vetted microarrays provide practical benefits, including lower per-sample cost (enabling larger cohorts/time-series), fixed probe content with embedded controls for rapid QC and cross-experiment comparability, shorter labeling-to-result turnaround for surveillance, and high reproducibility with standardized pipelines that limit between-run variability [5,7]. In pathogen surveillance scenarios (viral and bacterial), rapid turnaround and cross-experiment comparability are essential; fixed-content arrays facilitate longitudinal tracking of sentinel cohorts and routine screening. Our data exemplify these strengths: spike-ins behaved as expected, dye bias was absent, technical replicates were equivalent, and microarray intensities agreed with RT-qPCR rankings within the control set [43].

To place the performance of our array into a broader transcriptomic context, we next compared its reference expression profile with available RNA-seq datasets generated from healthy, non-infected common carp. Published transcriptomes, including those focused on ovarian cell composition [49], SSR variation in immune genes [50], transcriptome assembly [51], and pigmentation pathways [52,53], cover biological processes distinct from those analyzed here. Nevertheless, these studies consistently report signatures of innate immune readiness, metabolic regulation, and cellular stress responses, which broadly align with the functional modules captured by our platform. Additional RNA-seq datasets from healthy carp further support these patterns. The multi-tissue de novo transcriptome of Ji et al. [13] showed broad representation of KEGG immune, signaling, and metabolic categories across healthy tissues. Large-scale profiling of 28 tissues by Das et al. [54] likewise revealed enrichment of innate-immune and stress-related pathways, including *NOD*-like receptor signaling, *MAPK* signaling, necroptosis, and phagosome pathways through associations between non-coding RNAs and their mRNA targets. Although centered on nutritional phenotypes, Chen et al. [55] reported that liver transcriptomes of healthy carp display strong cytokine, chemokine, and oxidoreductase activity, indicating that immune-regulatory and redox pathways feature prominently even under non-challenged conditions. The multi-tissue atlas of Kolder et al. [56] further demonstrated that immune specialization is a consistent characteristic of carp lymphoid tissues. Collectively, these RNA-seq datasets highlight the same broad functional axes: innate immune readiness, signaling, metabolic regulation, and oxidative-stress responses that dominate the head kidney expression profile captured by our 8 × 60 K array, although none of them provide gene-level expression information for healthy head kidney or report the hemoglobin, complement, or *PRDX* paralogs resolved by our microarray. Among immune-focused datasets, the kidney transcriptome of [50] provides the most relevant point of comparison. Their study, also conducted on healthy fish, documented extensive repertoires of *TLR, NLR, RLR*, cytokine, chemokine, and complement paralogs consistent with the expanded immune gene families characteristic of allotetraploid cyprinids. Our findings of strong innate immune signatures in healthy head kidneys agree with this immunogenomic profile and underscore the immunological specialization of pronephros under homeostatic conditions. A contrasting perspective comes from Neave et al. [57], who analyzed the head kidney transcriptome during CyHV-3 infection rather than in healthy conditions. Acute infection induced strong activation of interferon signaling, inflammatory mediators, oxidative stress enzymes, and genes such as *iNOS*, *IRG1*, myeloperoxidase, and latexin, whereas mock-infected controls showed minimal activation. The baseline expression pattern in our dataset closely resembles this non-activated mock state: we observed no interferon-driven transcriptional reprogramming, no acute-phase inflammation, and no stress signatures characteristic of infection. Instead, homeostatic modules dominated, including heme metabolism, oxygen transport, protease regulation, and *PRDX*/thioredoxin-dependent antioxidant pathways. Importantly, none of the existing RNA-seq datasets from healthy carp provide a comprehensive reference profile of head kidney comparable to that presented here. Our microarray therefore fills an underrepresented area of carp transcriptomics by characterizing gene families, including hematopoietic and redox-regulatory modules that remain insufficiently captured in earlier RNA-seq studies of healthy fish.

As with any hybridization platform, dynamic range is narrower than RNA-seq, probes cannot capture unannotated isoforms, and residual cross-hybridization is possible. We mitigated these constraints by collapsing to one probe per gene, favoring 3′ regions compatible with T7/oligo(dT) protocols, and running multiple QC layers (spike-ins, stringency probes, FE flags). Interpretation emphasized ortholog-level enrichment against *Danio rerio*, leveraging the dense zebrafish annotation to avoid carp-specific annotation sparsity. Future work may increase coverage for selected pathways (e.g., three-probe designs in a 4 × 180 K format) or combine targeted RNA-seq for discovery with the microarray for routine monitoring. Future work will include validation in viral and bacterial challenge experiments and the development of targeted panels (e.g., ISG and chemokine modules) for rapid outbreak monitoring.

## 4. Materials and Methods

### 4.1. Reference Genome and Assembly

Probe design and microarray construction in this study were based on the *C. carpio* L. SPL01 reference assembly ASM1834038v1 with GenBank accession GCA_018340385.1, RefSeq accession GCF_018340385.1, and BioProjects PRJNA682709 and PRJNA745992. The SPL01 is a monoisolate registered in 2021 by the Chinese Academy of Fishery Sciences and is designated as agriculturally relevant. The assembly is a nuclear genome of about 1.68 Gb resolved into 50 chromosomes. Reference sequences in FASTA format and annotations in GFF/GTF format were obtained from NCBI Datasets/Assembly. SPL01 has been used in recent RNA-seq and comparative studies and is commonly used as the nuclear reference for common carp [46,47,58,59,60].

### 4.2. Custom Microarray Design

A custom gene-expression microarray for *C. carpio* L. was designed in the Agilent eArray environment (Agilent Technologies, Santa Clara, CA, USA) using the C_carpio_ASM1834038v1 resource under design ID 086984. The platform employed an 8 × 60 K format per slide with randomized feature layout and the IS-62976-8_V2_60kby8_GX_EQC_201000210 control grid. Each slide carried eight subarrays. In total, 62,976 features were specified, including 1319 control probes. The biological probe set consisted of 50,770 unique probes. A subset of 20 biological probes was used as technical replicate probes which were represented 10 times across the array. Feature placement used randomized assignment of biological probes across subarrays to minimize spatial bias. The probes were 60 nucleotides in length. Probes were prepared using the Base Composition design method, which ranks candidates by base-composition scoring with a positional bias toward the 3′ end [61]. Prior to probe selection, vector sequences and repetitive/low-complexity regions were masked, and masked segments were ignored during candidate evaluation. To minimize cross-hybridization, eArray screened candidates against the common carp transcriptome similarity database; in the final design, 5356 probes were flagged as having cross-hybridization potential (Supplementary Table S8).

### 4.3. Transcript Selection and Probe Placement

The initial dataset comprised 91,197 transcripts. Given extensive alternative splicing, transcript records from the same loci were collapsed to the gene level, yielding 51,096 gene models. Of the 51,096 submitted sequences, probes were not designed for a small subset due to sequence constraints (too short, *n* = 13; duplicate, *n* = 293; repeat/vector-masked, *n* = 16; probe could not be designed, *n* = 4), resulting in 50,770 biological probes. Analysis of the GCF_018340385.1 assembly showed that individual genes often have two to at least fourteen isoforms with distinct exon composition, increasing the risk of cross-hybridization for probes targeting closely related isoforms. To balance specificity with coverage, the design followed an established strategy: the longest available transcript was selected as the representative for each gene, and a single probe was positioned within the region 1000 bp from the 3′end- to match methods that capture mRNA from the poly(A) tail (Agilent eArray Help, available from https://earray.chem.agilent.com/earray/helppages/set_up_a_ge_probe_design_job.htm (accessed on 16 November 2025)). Relative to a one-probe-per-transcript approach without isoform collapsing, this gene-level reduction decreased the projected proportion of potentially cross-hybridizing probes from approximately 40% to approximately 10%. This strategy enabled a one-probe-per-gene design within the 8 × 60 K format while preserving the option to increase density to as many as three probes per gene in a 4 × 180 K format, if required in future iterations. All raw files, processed expression matrices, and platform annotations are openly available on Zenodo at https://doi.org/10.5281/zenodo.17360611 upon publication [62].

### 4.4. Healthy Fish and Tissue Collection

Healthy common carp weighing 90 g were acclimated to laboratory conditions for 2 weeks. A randomly selected subset of fish was screened for parasitic and bacterial agents, with negative results. Throughout acclimation and subsequent procedures, water temperature was maintained at 14 °C, dissolved oxygen at 6 mg/L, flow at 15 L/h, and a 12:12 h light-dark cycle was used. Fish were fed a complete diet formulated for common carp. No health disorders or mortality were observed during acclimation. After the acclimation period, fish were monitored daily for an additional 3 weeks for clinical signs; no health abnormalities or deaths were recorded during the entire observation period. Following euthanasia, internal organs were collected aseptically for downstream analyses. Kidneys were routinely excised on ice, transferred to RNAlater (Invitrogen, Waltham, MA, USA), placed in sterile vessels, and immediately stored at −80 °C until processing. All procedures involving fish complied with Polish law (Act of 15 January 2015 on the protection of animals used for scientific or educational purposes) and the 3Rs principles, and were approved by the Local Ethics Commission [permit no. 76/2021].

### 4.5. RNA Extraction

Total RNA was isolated from head kidney tissue using the RNeasy Mini Kit (Qiagen, Hilden, Germany) according to the manufacturer’s instructions, with homogenization in a bead-mill FastPrep-24 5G (MP Biomedicals, Santa Ana, CA, USA). For each sample, 30 mg of tissue was processed in 600 µL of RLT buffer. RNA quantity and purity (A260/280 and A260/230 ratios) were assessed spectrophotometrically using a NanoPhotometer (Implen, Munich, Germany). RNA integrity was determined with an Agilent 2100 Bioanalyzer (Agilent Technologies, Santa Clara, CA, USA) using the RNA 6000 Nano Kit (Agilent Technologies, Santa Clara, CA, USA). Only samples with RIN ≥ 9.0 and without secondary degradation peaks were retained for further analyses; preparations failing purity or integrity criteria were excluded and re-extracted. Eight biological replicates (*n* = 8 fish) were included for the healthy fish group, and one technical replication was performed at the RNA stage. For each fish, a 1 µg RNA aliquot was prepared. The eight aliquots were combined to create a control pool (Isolation 1), and an equivalent replicate was prepared from the same eight samples to create a control pool (Isolation 2). Both pools were used in subsequent analyses.

### 4.6. Two-Color Microarray Labeling and Hybridization

Total RNA was processed using the Low Input Quick Amp Labeling Kit (Agilent Technologies, Santa Clara, CA, USA) according to the manufacturer’s instructions for two-color gene-expression arrays (Figure 1). Briefly, spike A assigned to Cy3, and spike B assigned to Cy5 were serially diluted as recommended for the input RNA amount and added to each sample prior to labeling. cDNA synthesis was performed with a T7-oligo(dT) primer and AffinityScript RNase Block, followed by heat inactivation per the kit protocol. In vitro transcription with the T7 RNA Polymerase Blend generated fluorescent antisense cRNA by incorporation of cyanine 3-CTP or cyanine 5-CTP, separate reaction mixes for each dye. Labeled cRNA was purified on RNeasy Mini spin columns, quantified spectrophotometrically (NanoPhotometer, Implen), and verified for specific activity against Agilent criteria prior to hybridization. Samples meeting the activity threshold of ≥6 pmol/µg cRNA for both Cy3 and Cy5 were advanced to hybridization. Dye assignment was fixed as Cy3 for pool A and Cy5 for pool B for all primary comparisons. Hybridization samples were prepared by fragmenting Cy3- and Cy5-labeled cRNA at 60 °C for 30 min in the presence of 10× Gene Expression Blocking Agent and 25× Fragmentation Buffer, immediately cooling on ice, and stopping the reaction with 2× Hi-RPM Hybridization Buffer. Arrays were hybridized in a rotating oven at 65 °C for 17 h and then washed twice using the Gene Expression Wash Buffer Kit (Agilent Technologies, Santa Clara, CA, USA) in accordance with the manufacturer’s specifications.

### 4.7. Scanning and Feature Extraction

Following hybridization, microarrays were washed sequentially in the two Gene Expression Wash Buffers and scanned at 3 µm resolution using the Agilent G3_GX_2color protocol on an Agilent G2505C scanner (Agilent Technologies, Santa Clara, CA, USA). Images were processed in Agilent Feature Extraction (FE) Software v12.0.3.1 (Agilent Technologies, Santa Clara, CA, USA) under the two-color gene-expression protocol GE2_1200_Jun14. The software performed automated grid alignment to the array design (with assessment via IsGoodGrid), computed spot intensities with local background correction and spatial de-trending, and applied within-array LOWESS (linear dye normalization) together with additive-multiplicative error modeling at the feature level. As part of quality control, FE reported standard metrics and flags, including IsWellAboveBG for both channels, IsSaturated, non-uniformity and outlier indicators (FeatureNonUniformOutlier, PopulationOutlier), the two-color noise metric DLRSpread, distributions and statistics for negative controls alongside the background used (gBGUsed, rBGUsed), performance of the E1A spike-in panel (observed-expected relationship, slope, correlation, and %CV), stringency probes (DCP) metrics, the percentage of spike-ins passing (%Spike-In passed), the proportion of non-uniform features (%Non-Uniform), replicate variability (replicate %CV), and basic signal-to-noise summaries. Upon completion, FE generated text output files containing background-corrected processed intensities for the green and red channels (gProcessedSignal, rProcessedSignal), which served as the starting point for downstream analyses described in subsequent sections.

### 4.8. Data Processing and Statistics

Agilent FE outputs were imported into GeneSpring 14.8 Multi-Omic Analysis (Agilent Technologies, Santa Clara, CA, USA) as a two-color gene-expression experiment, using gProcessedSignal and rProcessedSignal as input intensities (background-corrected in FE). Upon import, intensities were log_2_-transformed, and subsequent analyses were carried out in log space. Following the FE workflow, within-array LOWESS dye normalization was applied during feature extraction; between-array harmonization was then performed in GeneSpring via baseline-to-median centering. Channel balance and array-level diagnostics were evaluated from log_2_(r/g) values using MA plots and distribution summaries. Quality filtering used FE-propagated flags and QC measures (e.g., non-uniformity, saturation, outlier status). Technical replicate spots were collapsed by robust averaging/median at the probe level. Where a gene was represented by multiple independent biological probes, a resistant consensus (median/robust average) was computed to obtain a single gene-level value. Probe-to-gene annotations followed the custom array design table used during platform construction. Differential expression was assessed with the moderated *t*-test (limma) as implemented in GeneSpring, with Benjamini–Hochberg false discovery rate (FDR) control. Unless stated otherwise for a specific contrast, statistical significance required FDR (q) ≤ 0.05 together with a fold-change threshold of |FC| ≥ 2.0.

### 4.9. Probe-Level QC for the Differentially Measured Probes

To distinguish true differential signals from potential x-hyb or low-intensity artifacts, we re-analyzed the raw Agilent Feature Extraction (FE) outputs for all 16 hybridizations (two technical spots per probe; 32 spots per probe). For the green (Cy3) channel we extracted: gMedianSignal, gBGMedianSignal, gBGPixSDev, gProcessedSignal, and FE quality flags (gIsWellAboveBG, gIsPosAndSignif, gIsFeatNonUnifOL, gIsFeatPopnOL, gIsSaturated). For every probe × array we computed the signal-to-noise ratio (SNR) as SNR_g_ = (gMedianSignal − gBGMedianSignal)/gBGPixSDev and summarized replicate spots by the median SNR (means were used for signal/background metrics). A probe was considered robustly detected on a given array if gIsWellAboveBG = 1 and SNR ≥ 3; values 1.5–3 were treated as cautionary and <1.5 as low-confidence. Arrays carrying any of the flags gIsFeatNonUnifOL = 1, gIsFeatPopnOL = 1, or gIsSaturated = 1 were marked and excluded from robustness claims for that probe. For each probe we then produced an across-array summary (median SNR, min–max SNR, mean gProcessedSignal, proportion of arrays with present calls, and any flags). Probes mapping to multi-gene families (e.g., globins) or to closely related paralogs (e.g., intelectin-like) were annotated a priori as higher x-hyb risk and interpreted together with the SNR/flag evidence.

### 4.10. Experimental Design

**Figure 5:**
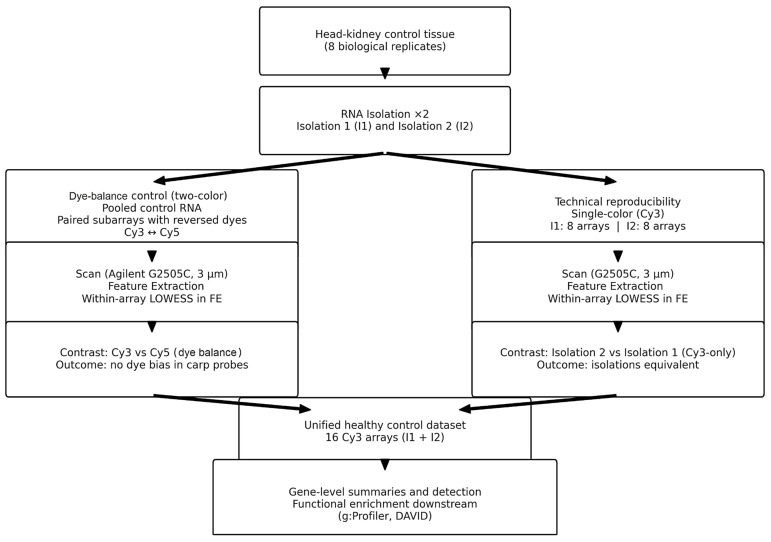
Experimental design and analysis pipeline.

For dye-balance control (self-self), the same pooled control RNA (isolation 1) was independently labeled with Cy3 and Cy5 and co-hybridized on three subarrays of a single Agilent 8 × 60 K slide, as described in Section 4.6, Section 4.7 and Section 4.8. For each subarray, the within-field Cy5-versus-Cy3 contrast was evaluated to assess and exclude systematic dye bias. These data were used only to verify dye balance and normalization (Figure 5). For technical reproducibility of RNA isolation (single channel), two independent RNA isolations from the same head kidney control pool (isolation 1 and isolation 2) were labeled exclusively with Cy3 and hybridized on eight subarrays per isolation (16 arrays total). The primary contrast compared Isolation 2 with Isolation 1, using each subarray as the experimental unit and analyzing the Cy3 channel only to isolate extraction-related variance from dye effects. After confirming the equivalence of the two isolations, the 16 Cy3 arrays were combined into a unified control dataset. No group contrast was defined; instead, gene-level summary metrics (signal distributions and detection rates) were computed, followed by functional annotation and enrichment analyses on the combined set.

### 4.11. Identification of C. carpio L.—Danio rerio Orthologs and Functional Enrichment Analysis

Orthologs of *C. carpio* L. genes to *Danio rerio* were identified using Ensembl (release 115). When a one-to-one match was not available, orthology assignments were verified or completed using OrthoDB v12.2, selecting *D. rerio* counterparts with conserved functional domains based on Ensembl and InterPro annotations. In total, 614 orthologs were retrieved and are listed in Supplementary Table S3. Functional enrichment analysis was performed using g:Profiler (g:GOSt) (https://biit.cs.ut.ee/gprofiler/gost (accessed on 15 September 2025); build *e113_eg59_p19_f6a03c19*) with *Danio rerio* (Zebrafish) as the reference organism [18]. Default parameters were applied (statistical domain scope: only annotated genes; significance threshold: g:SCS, α = 0.05). The analysis included annotations from Gene Ontology: molecular function (MF), cellular component (CC), biological process (BP), and pathway databases KEGG, Reactome, and WikiPathways. To validate these results, a second functional enrichment analysis was conducted using DAVID Bioinformatics Resources 2021 (https://davidbioinformatics.nih.gov/home.jsp (accessed on 12 September 2025), employing the Functional Annotation Clustering module [17]. Genes showing significant differential expression were analyzed, and associated annotations (GO_BP, GO_MF, GO_CC, KEGG, Reactome, InterPro, and UniProt Keywords) were grouped into functionally related clusters based on semantic similarity. The statistical significance of each enrichment term was assessed using the EASE score (a modified Fisher’s exact test), with *p*-values adjusted for multiple testing by the Benjamini–Hochberg false discovery rate (FDR) correction. Results were considered statistically significant at *Benjamini* ≤ 0.05. For each cluster, an Enrichment Score (ES) was calculated as the negative logarithm of the geometric mean of the constituent term *p*-values (ES = −log_10_(mean *p*-value)), with clusters showing ES ≥ 1.3 regarded as significantly enriched (approximately corresponding to *p* < 0.05).

### 4.12. RT-qPCR Assays

Primer performance for *ACTB1* (reference) and targets *SAA*, *CCL19*, *CD209*, *MMP9*, and *CXCL8a* were evaluated on head kidney cDNA using 10-fold serial dilutions of pooled RNA-derived cDNA (100, 10, and 1 ng input per reaction). Amplification efficiencies (E, %) were calculated from standard-curve slopes as E = (10^−1/slope^ − 1) × 100%; amplicon specificity was verified by single-peak melt curves and agarose gel electrophoresis. Primer sequences are listed in Table 1.

For Ct measurements, pooled RNA (1.5 µg) was DNase-treated and reverse-transcribed (NG dART RT Kit; 47 °C, 50 min). qPCR was run on a Rotor-Gene Q in 25 µL reactions containing QuantiTect SYBR Green Master Mix, primers at 0.4 µM each (final), and 80 ng cDNA. Cycling: 95 °C for 5 min; 40 cycles of 95 °C for 15 s and 60 °C for 30 s; followed by a melt curve (72–95 °C, 0.5 °C increments). Each gene was assayed in technical triplicates. Expression values for correlation with the microarray were computed as −ΔCt, where ΔCt = Ct_gene_ − Ct_ACTB1_.

## 5. Conclusions

In summary, the platform provides a standardized gene-expression tool that applies current carp genome resources via an oligonucleotide microarray. The healthy head kidney transcriptome shows enrichment for erythropoiesis, oxygen transport, redox buffering, protease regulation, and secreted humoral factors, consistent with a homeostatic hematopoietic-immune organ in teleosts. With gene-level probe design, ortholog-guided interpretation, and QC procedures, the array supports hypothesis-driven aquaculture studies, routine health surveillance, and cross-study meta-analysis. RNA-seq is suited to discovery and isoform-level questions, whereas microarrays are suited to large-scale, rapid, standardized gene-level measurements across many samples. Importantly, the microarray platform offers a robust baseline transcriptomic reference for interpreting immune and physiological responses during pathogen exposure or environmental stress. Its compatibility with existing zebrafish annotations enables deeper biological interpretation, particularly for immunity-, hematopoiesis-, and redox-related pathways. Future studies employing viral and bacterial challenge models, as well as expanded probe sets or targeted diagnostic panels, will further enhance the utility of this platform for aquaculture research and health monitoring.

## Figures and Tables

**Table 1:** Primer sequences used for gene-expression analysis in common carp. For each gene, forward (F) and reverse (R) primers are shown with melting temperatures (Tm, °C). All primers were designed in Primer3 against Ensembl *C. carpio* L. reference Cypcar_WagV4.0, positioned within exons to ensure specific amplification of target transcripts.

Gene	Primer	Sequence (5′→3′)	Tm (°C)
*MMP9*	2732R	ACGCCAGGCAAATGATTTCAC	52.4
	2617F	TGTATTGACCCCACTCACATGT	53.0
*CXCL8a*	481R	ACCCATCGGTACAGCTTGAAA	52.4
	391F	AAAGCCCCATGAATGTCTGG	51.8
*CD209*	879R	CCCATTTCCACACTCCCTCA	53.8
	769F	GCCACCAACAACATCACACA	51.8
*SAA*	428R	GTCTGTAGCGGTTGGGGTTA	53.8
	316F	TGATGGAAGAGAGGCTCTGC	53.8
*CCL19*	730R	TGGGAACATCAGACAACAAGGA	53.0
	627F	GGCAGCTGATGTAGTCTTCG	53.8
*ACTB1*	198F	GGTTTTGCTGGAGATGATGC	51.8
	471R	CTGTTGGCTTTGGGATTGAG	51.8
*CgGluc* *	162F	ACTGCGAGTGGAGACACATGAT	54.8
	230R	TCAGGTGTGGAGCGGACAT	53.2

*—Ouyang et al., Vet. Res., 2013 [63].

## Data Availability

All the microarray dataset for healthy *C. carpio* L. kidney tissue is available are openly available on Zenodo at https://doi.org/10.5281/zenodo.17360611.

## Supplementary Materials

The following supporting information can be downloaded at https://www.mdpi.com/article/10.3390/ijms262311240/s1.

## Abbreviations

ACTB1—β-actin 1 (reference gene); BP—Gene Ontology: Biological Process; BH—Benjamini–Hochberg (false discovery rate control); CC—Gene Ontology: Cellular Component; cDNA—complementary DNA; cRNA—complementary RNA; Cy3/Cy5—cyanine 3/cyanine 5 fluorescent dyes; DAVID—Database for Annotation, Visualization and Integrated Discovery; DCP—Agilent stringency control probes (DCP series); DLRSpread—derivative log ratio spread (two-color noise metric, Agilent FE); EASE—Expression Analysis Systematic Explorer (modified Fisher’s exact test in DAVID); E1A—adenovirus E1A spike-in controls (Agilent); ES—enrichment score (DAVID); FE—Feature Extraction (Agilent software); FDR—false discovery rate; FC/logFC—fold change/logarithm of fold change; GFF/GTF—General Feature Format/Gene Transfer Format; GO—Gene Ontology; g:GOSt—g:Profiler Gene Ontology Statistics module; HB/HBA/HBB—hemoglobin (α/β subunits; gene symbols as used); H_2_O_2_—hydrogen peroxide; ISG—interferon-stimulated gene(s); IVT—in vitro transcription; KEGG—Kyoto Encyclopedia of Genes and Genomes; LOWESS—locally weighted scatterplot smoothing; MA plot—log ratio (M) versus average intensity (A) plot; MF—Gene Ontology: Molecular Function; miRNA—microRNA; MIAME—Minimum Information About a Microarray Experiment; MIP—major intrinsic protein (aquaporin family); ncRNA—non-coding RNA; ODT/oligo(dT)—oligo(deoxythymidine) primer; PBS—phosphate-buffered saline (only if used in figures/protocols; delete if not present); PRDX—peroxiredoxin (gene family); QC—quality control; qPCR/RT-qPCR—quantitative PCR/reverse-transcription quantitative PCR; RIN—RNA Integrity Number; RNP—ribonucleoprotein; RNA-seq—RNA sequencing; RT—reverse transcription; *SAA*—serum amyloid A; SPL01—*Cyprinus carpio* L. nuclear reference genome assembly (ASM1834038v1); TF—transcription factor; Tm—melting temperature; T7—bacteriophage T7 RNA polymerase; WP—WikiPathways; ΔCt/−ΔCt—delta-Ct/negative delta-Ct (qPCR normalization/abundance metric).
