# Supplementary figures and images for: Transcriptional Profiling of Common Carp: A Microarray-Based Framework for Aquaculture Research
Source: Int J Mol Sci. 2025 Nov 21;26(23):11240. doi: 10.3390/ijms262311240 (PMC12692531; doi:10.3390/ijms262311240)

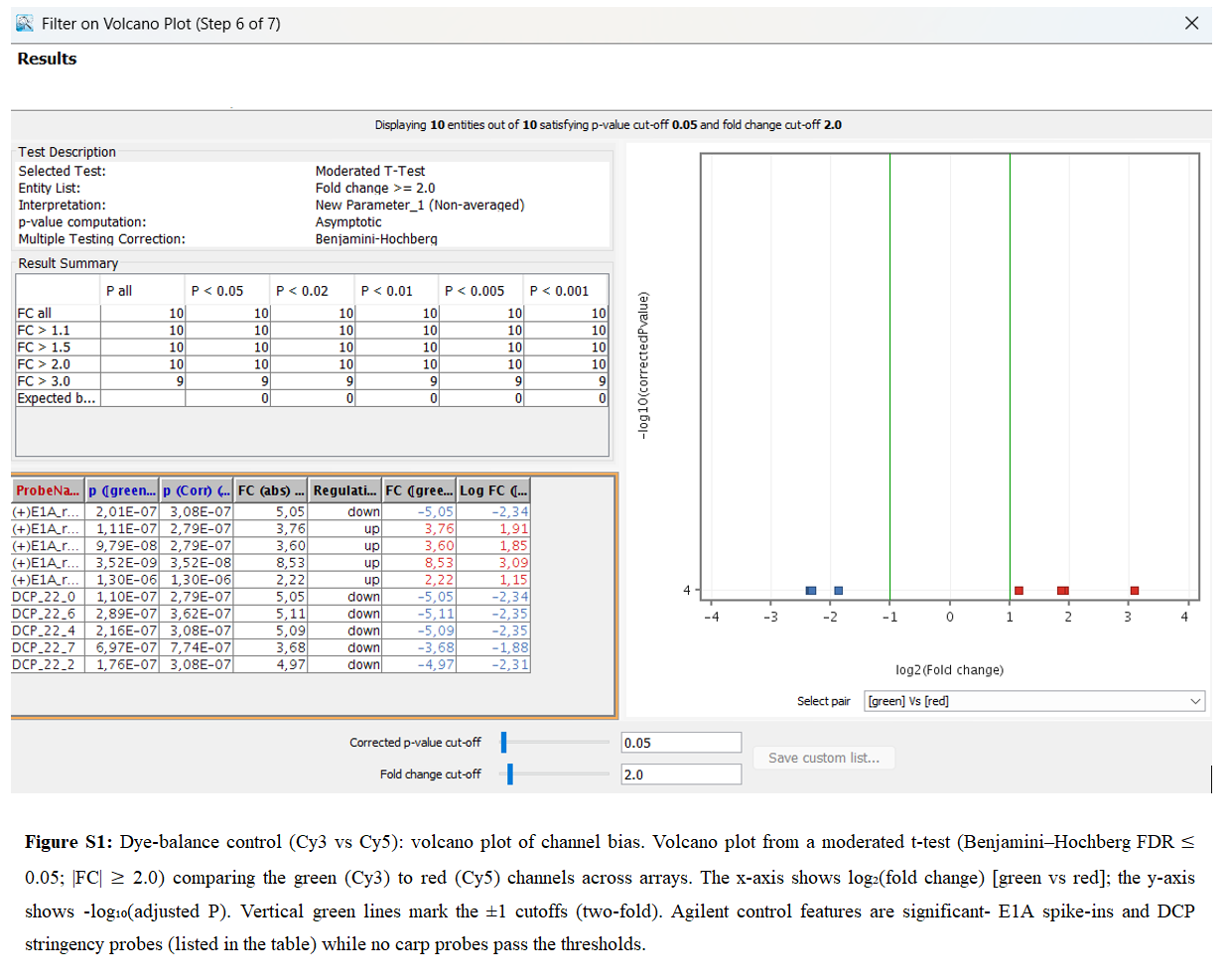

Supplement: Supplementary file 1 [file ijms-26-11240-s001.zip › Supplementary Figure S1.png]

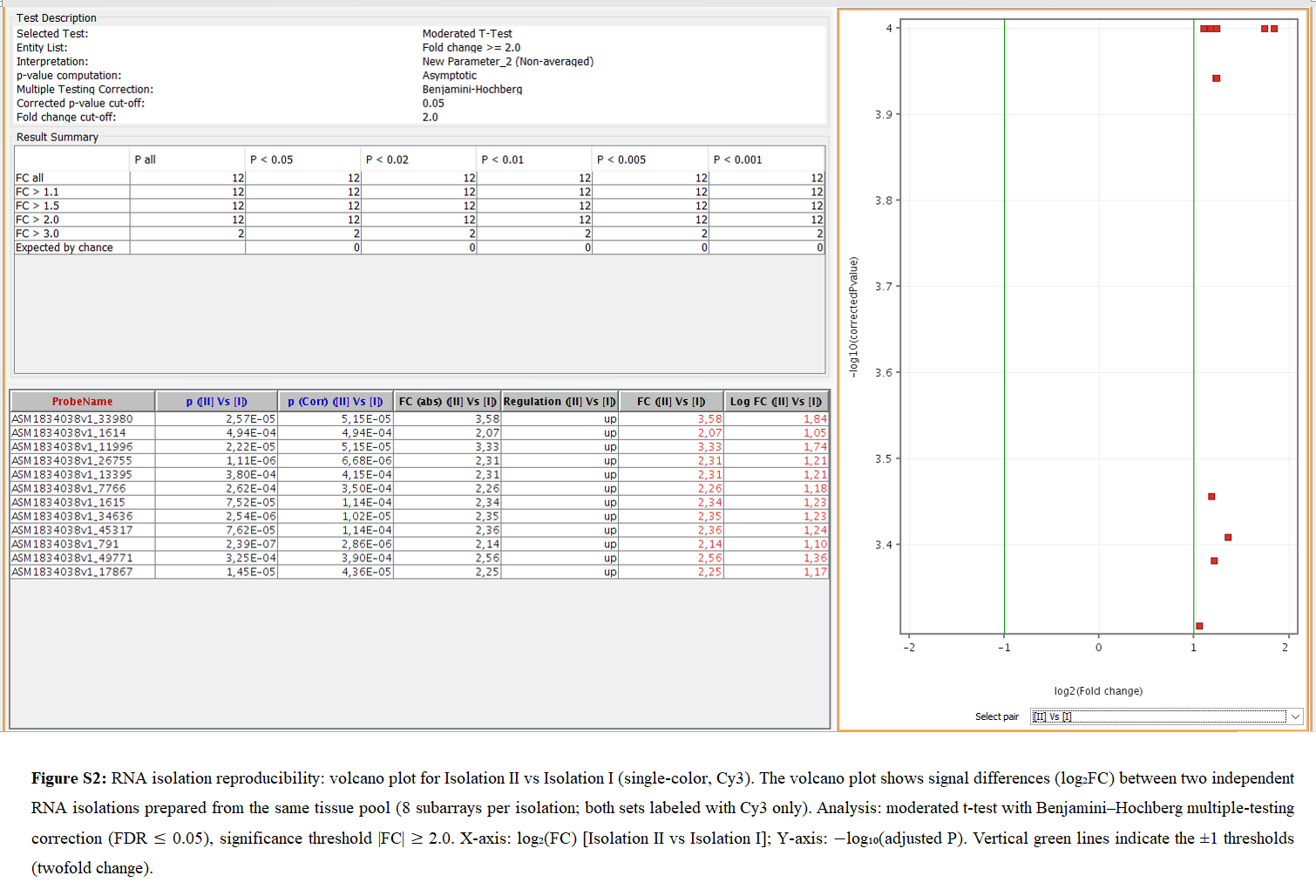

Supplement: Supplementary file 1 [file ijms-26-11240-s001.zip › Supplementary Figure S2.png]

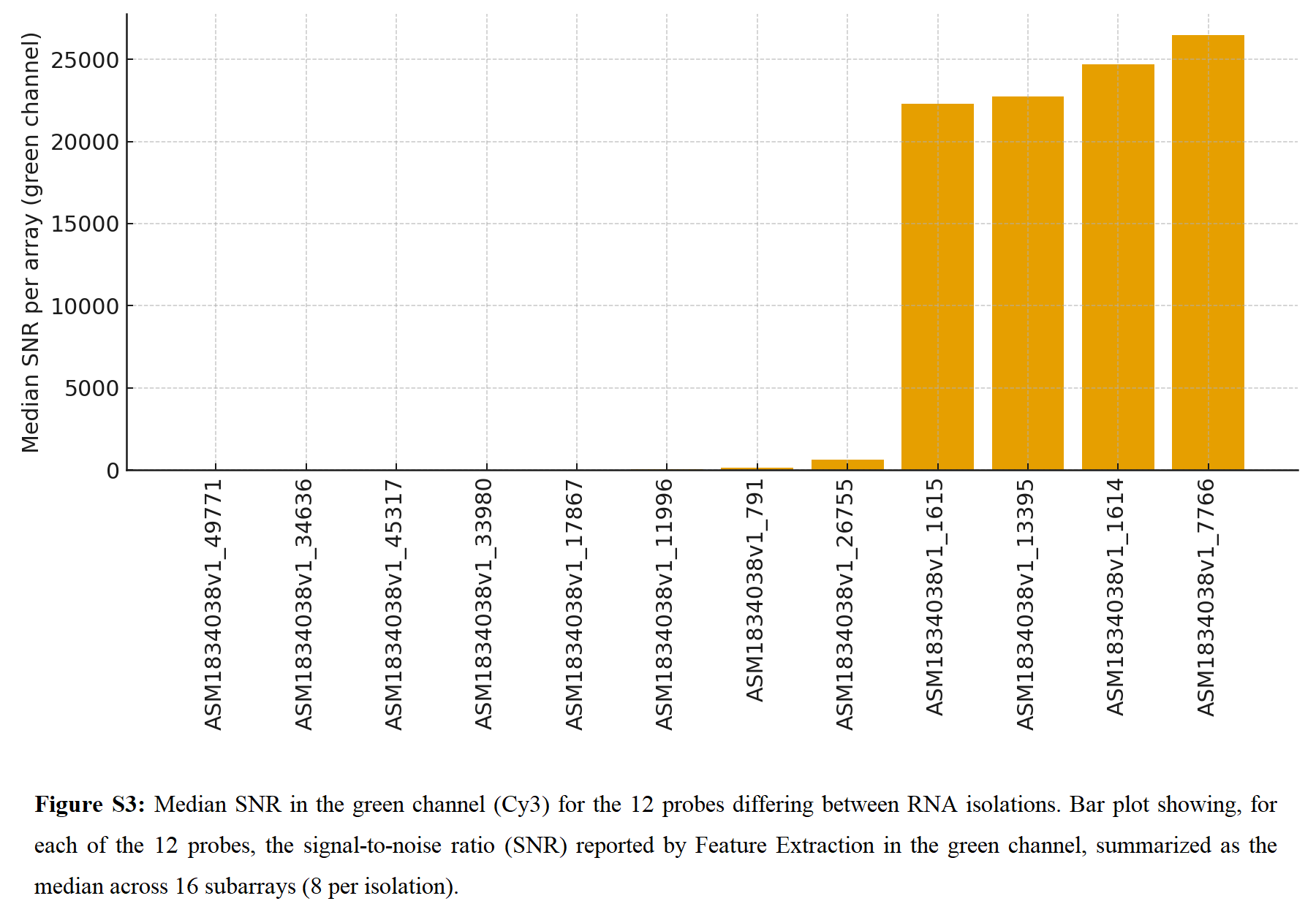

Supplement: Supplementary file 1 [file ijms-26-11240-s001.zip › Supplementary Figure S3.png]
